# Heart Failure Revealing Occult Rheumatoid Aortitis Presenting as a Giant Ascending Aortic Aneurysm With Functional Aortic Regurgitation: A Case Report

**DOI:** 10.7759/cureus.106014

**Published:** 2026-03-28

**Authors:** Mehdi Moujahid, Hafsa Erregui, Hicham Faliouni, Zouhair Lakhal, Aatif Benyass

**Affiliations:** 1 Cardiology, Mohammed V Military Instruction Hospital, Rabat, MAR

**Keywords:** aortic regurgitation, aortitis, ascending aortic aneurysm, heart failure, inflammatory aortopathy, rheumatoid arthritis

## Abstract

Rheumatoid arthritis (RA) is a systemic inflammatory disease that can rarely involve the aorta and remain clinically silent until severe complications occur. We report the case of an 80-year-old woman with no cardiovascular risk factors who presented with acute left-sided heart failure that led to the diagnosis of previously unrecognized rheumatoid arthritis. Transthoracic echocardiography demonstrated global left ventricular hypokinesia with a left ventricular ejection fraction of 34%, a structurally preserved tricuspid aortic valve, and moderate-to-severe functional aortic regurgitation related to ascending aortic dilation. The ascending aorta was markedly dilated, predominantly at the sinotubular junction and tubular segment. Computed tomography confirmed a giant ascending aortic aneurysm measuring 6.2 cm and revealed additional dilation of the right subclavian artery, suggesting multifocal large-vessel involvement. Laboratory investigations showed elevated inflammatory markers, and immunological testing supported the diagnosis of rheumatoid arthritis. These findings were highly suggestive of an underlying inflammatory aortopathy, although a definitive causal relationship could not be established. This case highlights that heart failure may be the presenting event revealing occult inflammatory aortopathy and underscores the importance of considering systemic inflammatory diseases in patients with unexplained aortic pathology, even in the absence of traditional cardiovascular risk factors.

## Introduction

Rheumatoid arthritis (RA) is a chronic systemic inflammatory disease primarily affecting synovial joints; however, extra-articular manifestations are common and significantly contribute to morbidity and mortality. Cardiovascular involvement represents a major determinant of prognosis in patients with RA and includes accelerated atherosclerosis, myocardial dysfunction, pericardial disease, and valvular abnormalities [1]. In contrast, inflammatory involvement of the aorta remains rare and frequently underrecognized, particularly in elderly patients. Although uncommon, aortic involvement in RA has been described in autopsy series, suggesting that subclinical disease may be underdiagnosed.

Aortitis is an uncommon but potentially life-threatening manifestation of systemic inflammatory diseases. Chronic inflammation of the aortic wall leads to progressive destruction of elastic fibers within the media, resulting in weakening of the vessel wall, aneurysm formation, and possible valvular dysfunction [2]. The spectrum of inflammatory aortic diseases is broad and includes large-vessel vasculitides as well as connective tissue disorders, among which rheumatoid arthritis represents a rare but well-documented cause [3]. In elderly patients, giant cell arteritis represents a more common cause of inflammatory aortitis and should be considered in the differential diagnosis.

Rheumatoid aortitis is infrequently diagnosed during life due to its insidious course and nonspecific clinical presentation. When present, it most commonly involves the ascending aorta and may lead to aneurysmal dilation and secondary aortic regurgitation, with heart failure as a potential initial manifestation [4]. These complications may occur even in the absence of traditional cardiovascular risk factors, further complicating the etiological diagnosis.

In addition, dilation of the ascending aorta can result in functional aortic regurgitation despite a structurally preserved aortic valve, through alteration of aortic geometry and impaired cusp coaptation [5]. The sinotubular junction corresponds to the transition between the aortic root and the tubular ascending aorta, while cusp coaptation refers to the proper closure of the aortic valve leaflets. Recognition of this mechanism is essential, as it differs from primary valvular disease and carries important diagnostic and therapeutic implications.

We present the case of an elderly woman with acute heart failure revealing a giant ascending aortic aneurysm with functional aortic regurgitation, leading to the diagnosis of previously unrecognized rheumatoid arthritis.

## Case presentation

An 80-year-old woman with no known cardiovascular risk factors was admitted to our cardiology department for the acute onset of dyspnea associated with orthopnea and progressive lower-limb edema. The patient reported progressive exertional dyspnea over the preceding weeks but had no prior history of ischemic heart disease, hypertension, diabetes mellitus, or previously diagnosed connective tissue disorder.

**Figure 1:**
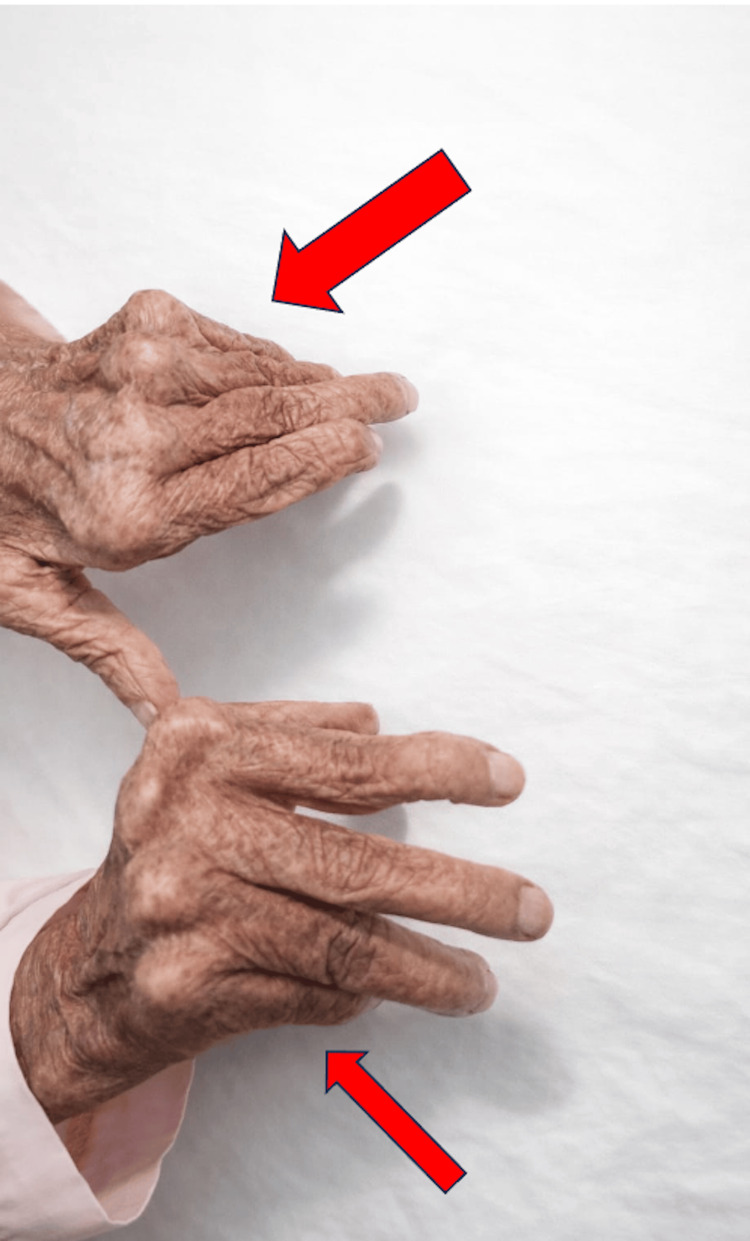
Clinical photograph demonstrating characteristic deformities of the hands consistent with long-standing rheumatoid arthritis, including metacarpophalangeal joint deformities and ulnar deviation.

On physical examination, the patient appeared dyspneic with clinical signs of left-sided heart failure. Blood pressure was 110/60 mmHg, and heart rate was 95 beats per minute. Cardiac auscultation revealed a diastolic murmur along the left sternal border. Peripheral examination showed bilateral pitting edema. Notably, inspection of the hands revealed ulnar deviation of the metacarpophalangeal joints associated with swan-neck deformities, consistent with long-standing rheumatoid arthritis (Figure 1).

**Figure 2:**
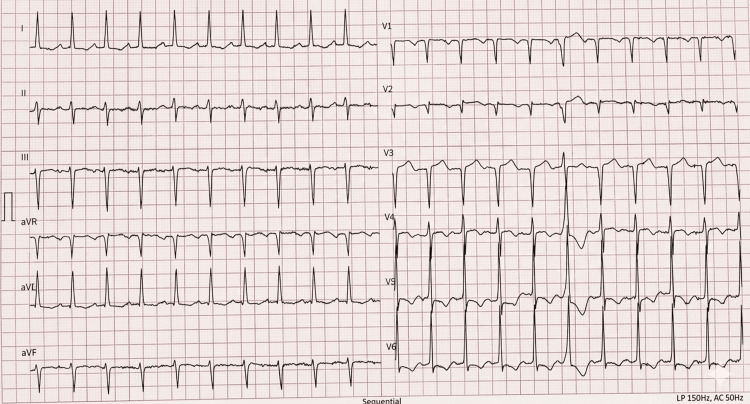
Electrocardiogram showing sinus rhythm with left ventricular hypertrophy and secondary repolarization abnormalities.

Electrocardiography demonstrated sinus rhythm with left ventricular hypertrophy and secondary repolarization abnormalities (strain pattern), including ST-segment depression in the high lateral leads (I and aVL) (Figure 2).

**Figure 3:**
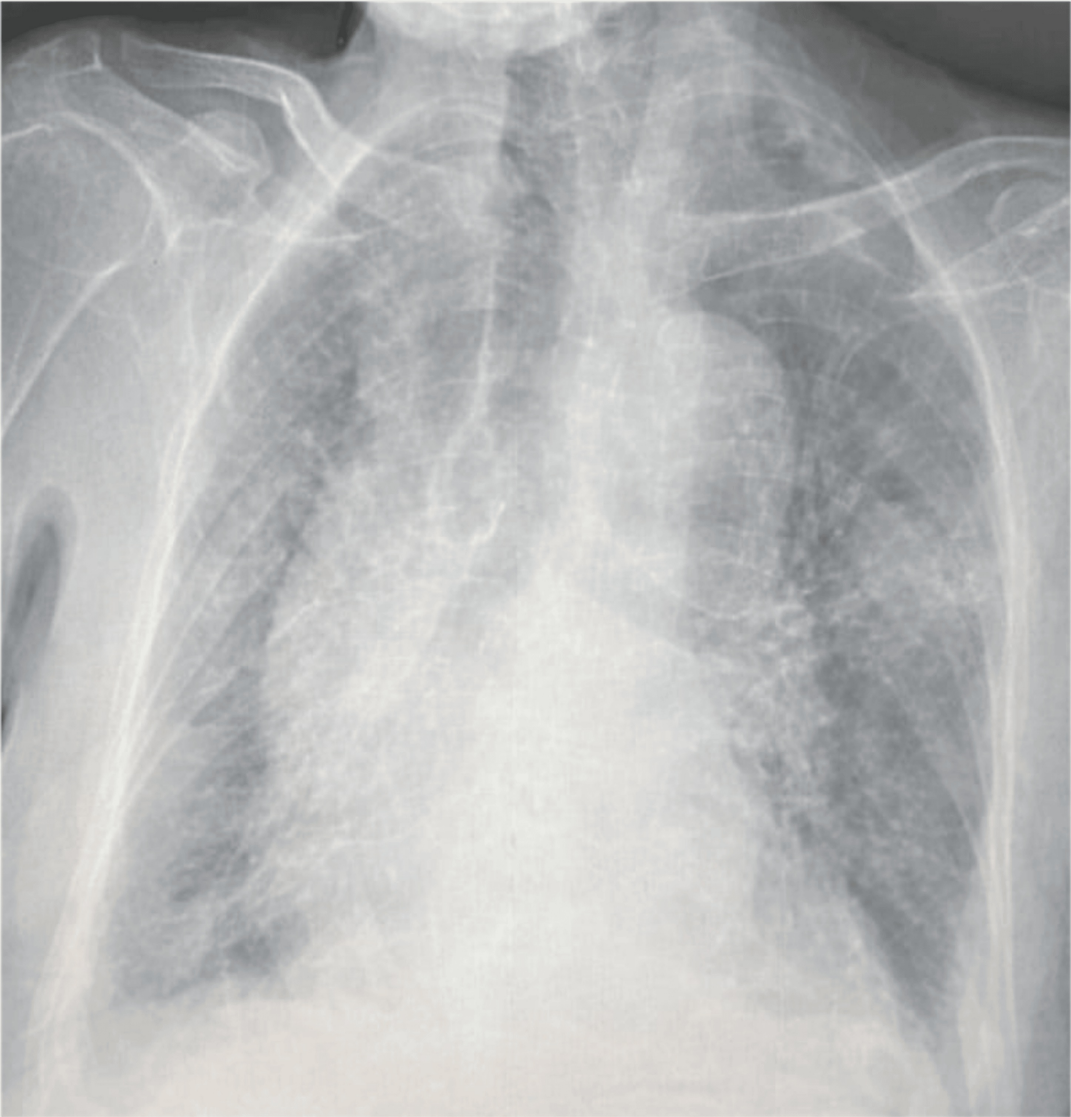
Chest radiograph demonstrating cardiomegaly associated with mediastinal widening and bilateral perihilar vascular congestion.

Chest radiography revealed cardiomegaly and mediastinal widening (Figure 3).

Transthoracic echocardiography demonstrated global left ventricular hypokinesia with a left ventricular ejection fraction of approximately 34%. Left ventricular systolic dysfunction was characterized by global hypokinesia without segmental wall motion abnormalities, making ischemic cardiomyopathy less likely. In this context, the reduced ejection fraction was considered multifactorial, primarily related to chronic volume overload due to aortic regurgitation and altered aortic compliance, while subclinical myocardial involvement related to systemic inflammation could not be excluded.

**Figure 4:**
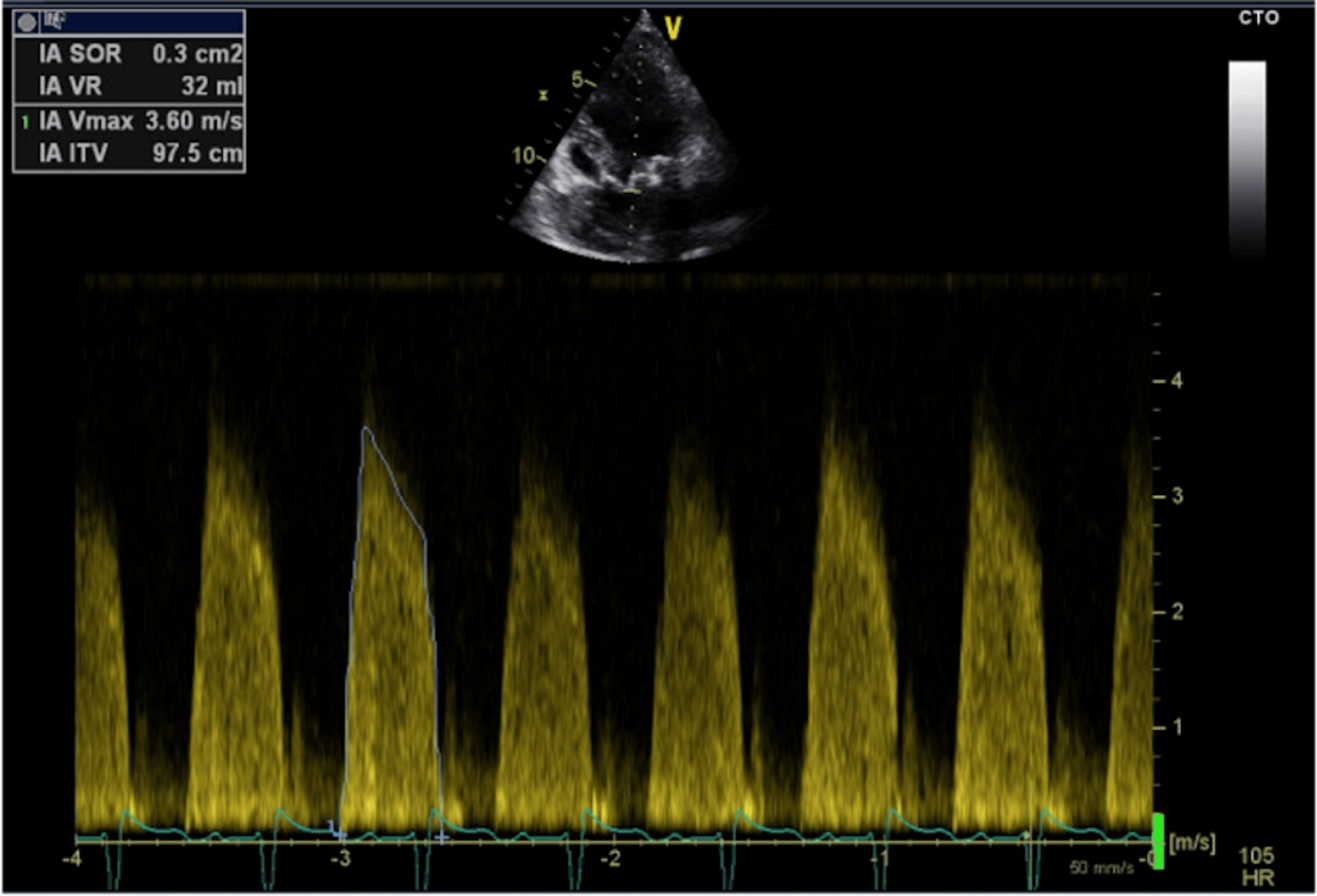
Continuous-wave Doppler echocardiography demonstrating aortic regurgitation with a dense holodiastolic regurgitant jet. Quantitative assessment showed an effective regurgitant orifice area of 0.3 cm² and a regurgitant volume of 32 mL, consistent with moderate-to-severe aortic regurgitation.

The aortic valve was tricuspid and structurally preserved. Moderate-to-severe aortic regurgitation was present on Doppler echocardiography, with quantitative assessment showing an effective regurgitant orifice area of 0.3 cm² and a regurgitant volume of 32 mL, supporting this grading (Figure 4).

**Figure 5:**
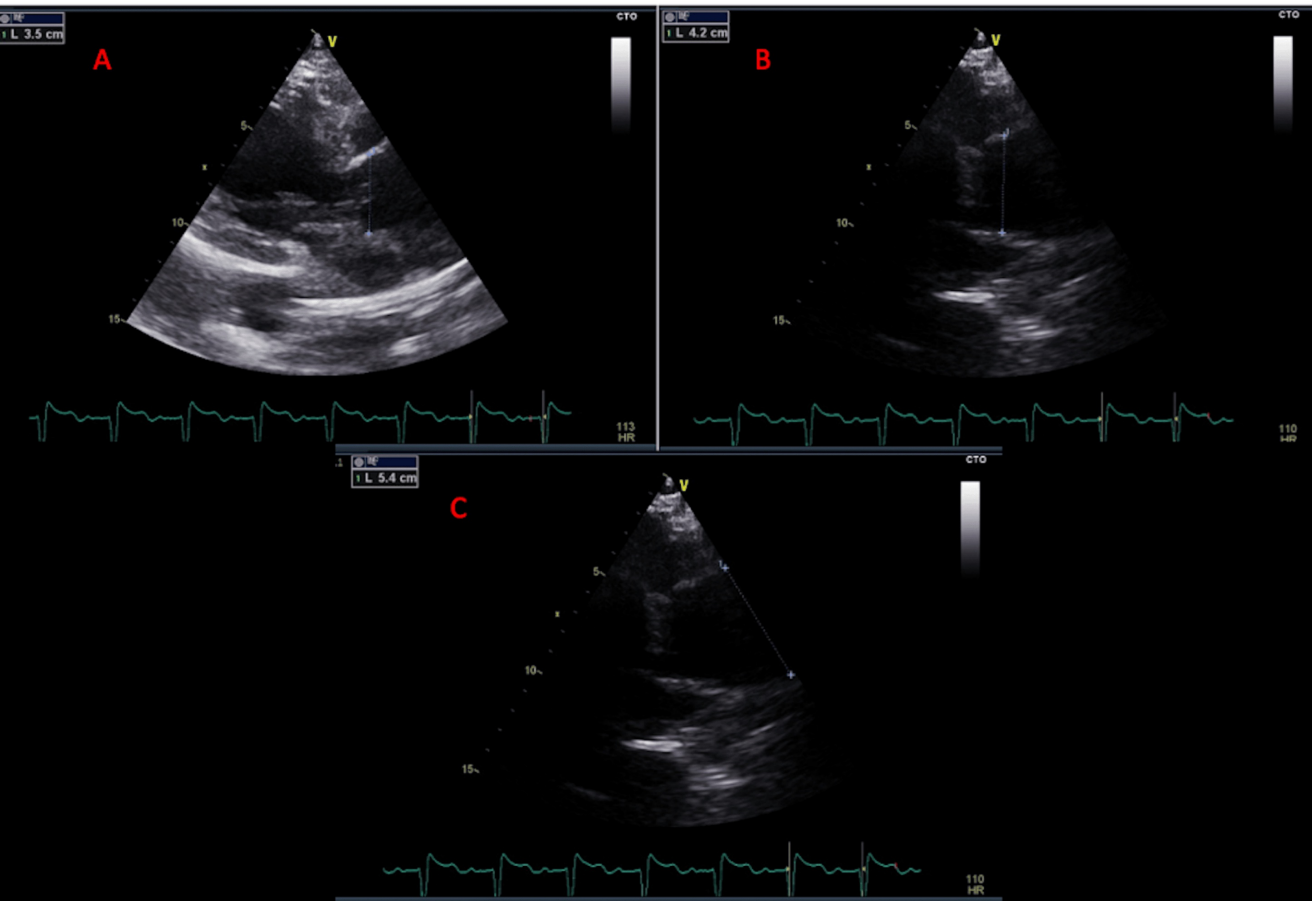
Transthoracic echocardiography demonstrating dilation of the proximal aorta with preserved tricuspid aortic valve morphology. (A) Parasternal long-axis view showing dilation at the level of the sinuses of Valsalva; (B) Measurement at the sinotubular junction demonstrating dilation; (C) Measurement of the tubular ascending aorta showing a maximal diameter of approximately 5.4 cm.

Detailed aortic assessment showed marked dilation of the ascending aorta, measuring 5.4 cm at the tubular segment, 4.2 cm at the sinotubular junction, and 3.5 cm at the level of the sinuses of Valsalva, while the aortic annulus remained within normal limits (Figure 5).

**Figure 6:**
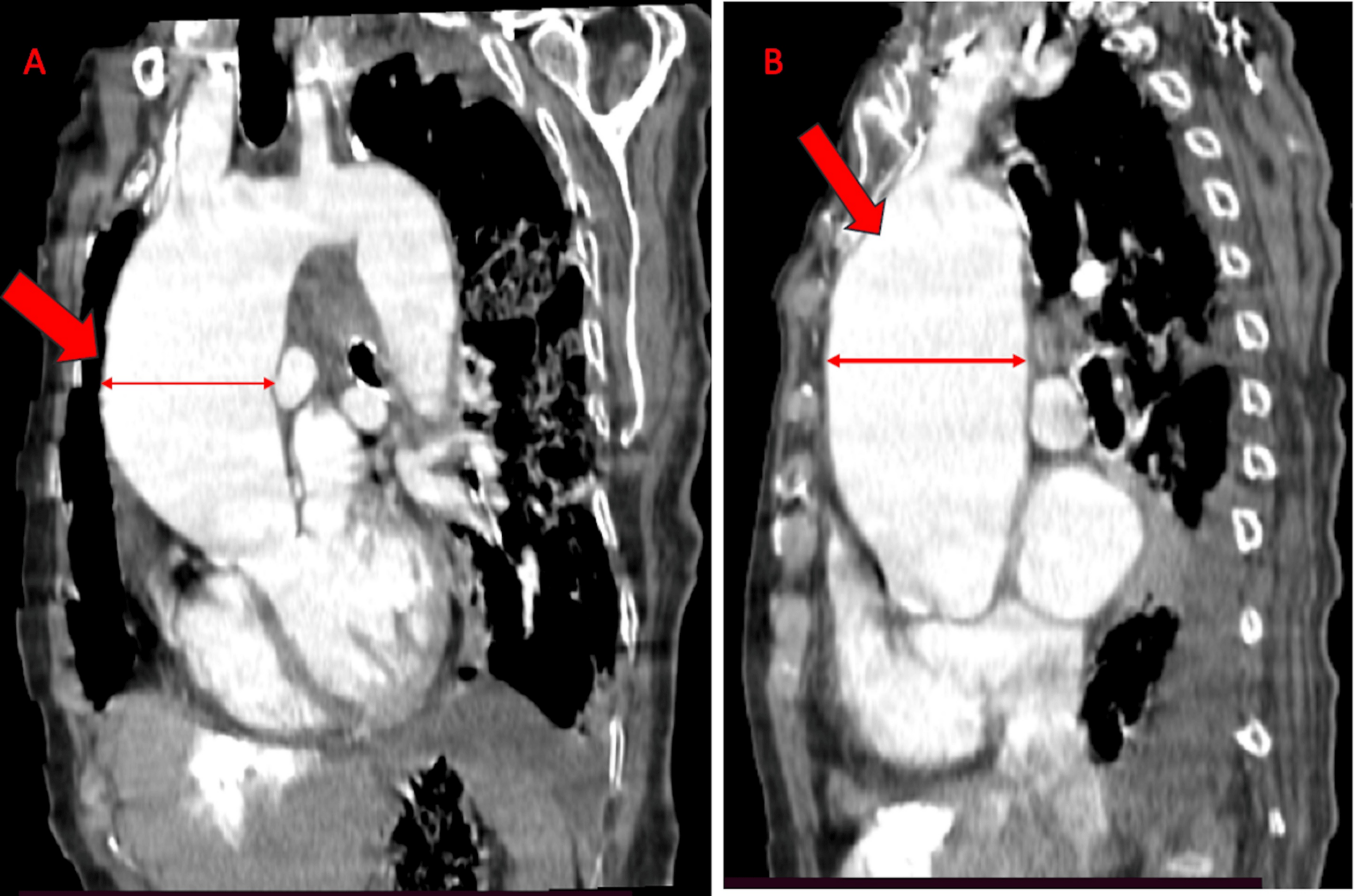
Contrast-enhanced computed tomography demonstrating a giant ascending aortic aneurysm. (A) Coronal reconstruction showing marked dilation of the ascending aorta; (B) Sagittal reconstruction illustrating the longitudinal extent of the aneurysm.

**Figure 7:**
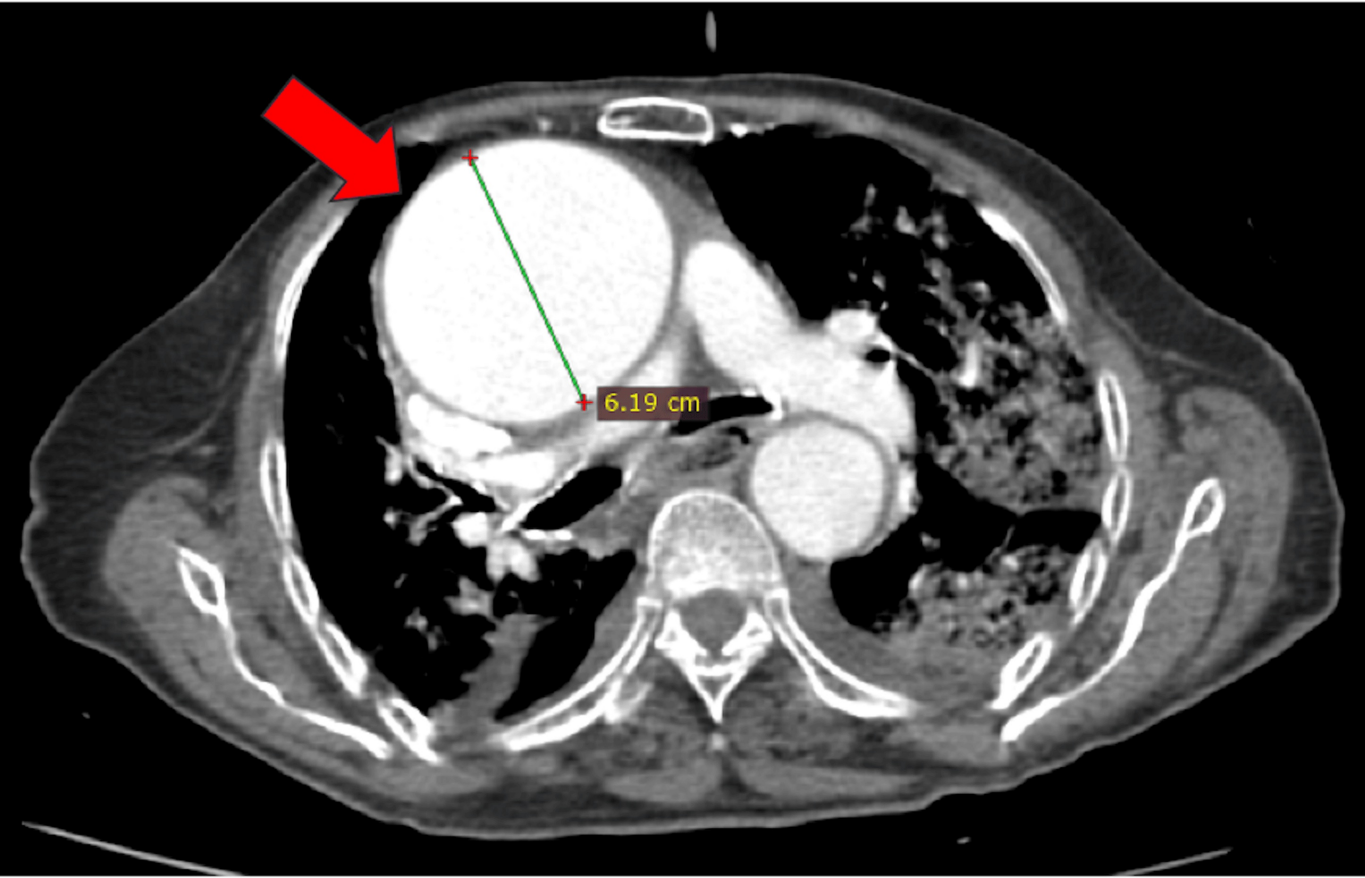
Axial contrast-enhanced computed tomography showing a giant ascending aortic aneurysm measuring approximately 6.2 cm in maximal transverse diameter.

Computed tomography confirmed a giant ascending aortic aneurysm measuring approximately 6.2 cm in diameter and revealed dilation of the right subclavian artery, supporting the presence of multifocal large-vessel involvement (Figure 6 and Figure 7).

Laboratory investigations showed elevated inflammatory markers, with C-reactive protein at 85 mg/L and erythrocyte sedimentation rate at 72 mm/h. Immunological testing revealed a rheumatoid factor level of 120 IU/mL and anti-cyclic citrullinated peptide antibodies at 180 IU/mL, while antineutrophil cytoplasmic antibodies were negative.

Based on the presence of inflammatory arthritis features, positive immunological markers, and atypical aortic involvement, the diagnosis of rheumatoid arthritis-associated inflammatory aortopathy was considered. The patient fulfilled the 2010 American College of Rheumatology (ACR)/European Alliance of Associations for Rheumatology (EULAR) classification criteria for rheumatoid arthritis, with a total score of seven points, including involvement of multiple small joints (three points), high-positive rheumatoid factor and anti-cyclic citrullinated peptide antibodies (three points), and elevated acute-phase reactants (one point).

The patient was treated with intravenous loop diuretics, oxygen therapy, and guideline-directed medical therapy for heart failure. A multidisciplinary discussion involving cardiology and rheumatology was conducted, and initiation of immunosuppressive therapy, including corticosteroids and potential disease-modifying antirheumatic drugs (DMARDs), was considered in the context of inflammatory aortopathy, with further management planned following surgical evaluation.

At short-term follow-up, the patient showed clinical improvement with resolution of congestion under medical therapy and remained under evaluation for definitive surgical management.

## Discussion

Rheumatoid arthritis is a systemic inflammatory disease in which cardiovascular involvement represents a major cause of morbidity and mortality. While accelerated atherosclerosis, myocardial dysfunction, and pericardial disease are well recognized, aortic involvement remains rare and frequently underdiagnosed, particularly in elderly patients [1,2]. RA-associated aortitis often follows a silent course and may only be revealed by complications such as aneurysm formation, valvular dysfunction, or heart failure.

The pathophysiology of RA-associated aortitis is characterized by chronic inflammation of the aortic wall, leading to progressive destruction of elastic fibers and weakening of the media. This process predisposes to aneurysmal dilation, most commonly involving the ascending aorta, and may secondarily affect aortic valve function [2,3]. In contrast to degenerative aortic disease, inflammatory aortopathy often presents with segmental or multifocal large-vessel involvement, as observed in our patient.

In the present case, several features argue against a degenerative etiology, including the absence of cardiovascular risk factors, preservation of a normal tricuspid aortic valve and annulus, and the presence of multifocal arterial involvement. These findings, together with characteristic hand deformities, elevated inflammatory markers, and positive rheumatoid immunological tests, strongly support the diagnosis of inflammatory aortopathy related to rheumatoid arthritis.

Other causes of inflammatory aortitis, including giant cell arteritis, Takayasu arteritis, and IgG4-related disease, were considered unlikely based on clinical presentation and laboratory findings.

The mechanism of aortic regurgitation in this patient was functional, resulting from altered aortic geometry rather than primary valvular leaflet disease. Dilation of the ascending aorta and sinotubular junction can impair cusp coaptation despite a preserved annulus, leading to regurgitation. The patient’s acute heart failure likely resulted from the combined effect of moderate-to-severe functional aortic regurgitation and left ventricular systolic dysfunction. In addition, subclinical myocardial involvement related to systemic inflammation may have contributed.

Multimodality imaging played a key role in the diagnostic process. Transthoracic echocardiography allowed assessment of ventricular function and valvular involvement, while computed tomography provided precise evaluation of aortic dimensions and the extent of large-vessel involvement. This complementary approach is essential for distinguishing inflammatory aortopathy from degenerative or other etiologies.

From a therapeutic perspective, early recognition of inflammatory aortopathy is crucial, as it has important implications for both medical and surgical management. In addition to guideline-directed therapy for heart failure, management may require immunomodulatory treatment and careful surgical planning for aortic repair, ideally within a multidisciplinary framework.

This case underscores several important clinical implications. Careful clinical examination remains essential in identifying signs of systemic inflammatory disease. Comprehensive aortic imaging is crucial to detect atypical patterns suggestive of inflammatory aortopathy. Multidisciplinary management is required to guide both medical and surgical decision-making.

This case is noteworthy because it illustrates heart failure, revealing a giant ascending aortic aneurysm with functional aortic regurgitation, leading to the diagnosis of previously unrecognized rheumatoid arthritis. Clinicians should maintain a high index of suspicion for systemic inflammatory diseases in patients presenting with unexplained aortic pathology, even in elderly individuals without traditional cardiovascular risk factors.

This case has several limitations. Comprehensive assessment of diastolic and right ventricular function was limited, and advanced imaging such as cardiac MRI or coronary evaluation was not performed. In addition, follow-up inflammatory markers were not available, limiting the assessment of response to therapy and the evolution of the inflammatory process.

## Conclusions

Rheumatoid arthritis can be associated with rare but severe cardiovascular manifestations, including inflammatory involvement of the aorta. This case illustrates how rheumatoid aortitis may remain clinically silent and be revealed by acute heart failure secondary to a giant ascending aortic aneurysm and moderate-to-severe functional aortic regurgitation. Early recognition of an underlying systemic inflammatory disorder is essential, as it has major implications for diagnosis, multidisciplinary management, surveillance, and timely surgical planning. Clinicians should maintain a high index of suspicion for inflammatory aortopathy in patients presenting with unexplained aortic dilation, even in the absence of traditional cardiovascular risk factors.
